# Elemental distribution in tissue components of N_2_-fixing nodules of *Psoralea pinnata* plants growing naturally in wetland and upland conditions in the Cape Fynbos of South Africa

**DOI:** 10.1007/s00709-013-0589-5

**Published:** 2013-12-24

**Authors:** Sheku A. Kanu, Alban D. Barnabas, Wojciech J. Przybylowicz, Jolanta Mesjasz-Przybylowicz, Felix D. Dakora

**Affiliations:** 1 Department of Crop Sciences, Tshwane University of Technology, Private Bag X680, Pretoria, 0001 South Africa; 2 Materials Research Department, iThemba LABS, PO Box 722, Somerset West, 7129 South Africa; 3 Chemistry Department, Tshwane University of Technology, Private Bag X680, Pretoria, 0001 South Africa

**Keywords:** Infected/uninfected cells, Leghaemoglobin, Nitrogenase, *Psoralea pinnata*, X-ray microanalysis, Elemental maps

## Abstract

There is little information on in situ distribution of nutrient elements in N_2_-fixing nodules. The aim of this study was to quantify elemental distribution in tissue components of N_2_-fixing nodules harvested from *Psoralea pinnata* plants grown naturally in wetland and upland conditions in the Cape Fynbos. The data obtained from particle-induced X-ray emission revealed the occurrence of 20 elements (Si, P, S, Cl, K, Ca, Ti, Mn, Fe, Ni, Cu, Zn, As, Br, Rb, Sr, Y, Zr, Mo and Ba) in nodule components. Although, in upland plants, the concentrations of S, Fe, Si, Mn and Cu showed a steady increase from the middle cortex to the medulla region of *P. pinnata* nodules, in wetland plants, only S, Fe and Mn showed an increase in concentration from the middle cortex to the bacteria-infected medulla of *P. pinnata* nodules. By contrast, the concentrations of Cl, K, Ca, Zn and Sr decreased from middle cortex to nodule medulla. The alkaline earth, alkali and transition elements Rb, Sr, Y and Zr, never before reported in N_2_-fixing nodules, were found to occur in root nodules of *P. pinnata* plants grown in both wetland and upland conditions.

## Introduction

Mineral nutrients are important for growth and cellular functioning of plants, microbes and their symbiotic interaction inside root nodules. Determinate N_2_-fixing nodules such as those of the Phaseoleae (e.g. cowpea and soybean) are characterized by the presence of an outer cortex, middle cortex, inner cortex (or “nodule parenchyma”, Van de Wiel et al. 1990) and a central medulla region (Frazer 1942; Dakora and Atkins 1989), which itself consists of infected and uninfected interstitial cells (Kaneko and Newcomb 1987; Webb and Newcomb 1987). In N_2_-fixing nodules, both the cortical and medulla components are interspersed by intercellular airspaces that serve as diffusional pathways for oxygen transport to respiring bacteroids in the infected cells (Dakora and Atkins 1989; Dakora and Atkins 1990a, b; Dakora and Atkins 1991; Sherrier et al. 2005).

Nitrogen fixation by bacteroids in infected cells of root nodules is energetically a very expensive process, requiring at least 6 ATP molecules generated by oxidative phosphorylation per 2e^−^ transferred to N_2_. Nitrogen fixation is thus an oxygen-demanding process. Paradoxically, however, oxygen is a potent inhibitor of nitrogenase activity, irreversibly inactivating both the Fe and MoFe proteins of the enzyme via oxidation of the metal-S centres (Robson and Postgate 1980) and by repression of nitrogenase synthesis (Shaw 1983). To avoid denaturation of nitrogenase enzyme, leghaemoglobin (Lb) mediates oxygen delivery at low concentrations to bacteroids inside N_2_-fixing nodules (Appleby 1969; Appleby 1984; Appleby 1992; Limpens et al. 2003; Ott et al. 2005; Jones et al. 2007). This oxygen-binding protein consists of a porphyrin moiety and heme (Fe) synthesized by bacteroids (Cutting and Schulman 1971; Godfrey and Dilworth, 1971; Dénarié et al. 1976). The Lb protein is localized in the cytoplasm and nuclei of both bacteria-infected and uninfected interstitial cells (VandenBosch and Newcomb 1988; Vivo et al. 1989), with four times more Lb concentration in the infected cells relative to uninfected cells (VandenBosch and Newcomb 1988), and more Fe in the cell cytosol compared with the peribacteroid membrane (Dart and Chandler 1971). About 20–25 % of Lb in N_2_-fixing nodules is oxygenated (Appleby 1984), and it is the oxidation/reduction reactions (ferrous to ferric) of Lb that delivers a free oxygen concentration of about 10 nM to respiring bacteroids in the infected cells (Appleby 1984).

Nodule formation and functioning in symbiotic legumes therefore has a heavy demand on mineral elements for both plant and bacterial growth, and metabolic functioning such as the synthesis of macromolecules. It is thus not surprising that a number of studies (Rennie and Debutz, 1986; George et al. 1993; Sparrow et al. 1995; Jensen 1997; Unkovich and Pate 2000) have established a higher root uptake and tissue accumulation of mineral nutrients by nodulated legumes when compared with non-N_2_-fixing species. For example, apart from their requirement for plant and bacterial growth, nutrient elements such as P is needed in extra concentrations for ATP synthesis in support of nitrogenase activity in root nodules, just as extra Fe is required for Lb biosynthesis and the formation of nitrogenase enzyme in nodules. Although a number of studies (Atkins et al. 1984; Singleton and van Kessel 1987; Johnson et al. 2001) have addressed the role of mineral nutrients in symbiotic establishment and nodule functioning, few have examined their distribution in components of N_2_-fixing nodules, especially in relation to nutritional physiology and tissue mineral metabolism.

Even though the metabolic roles of various minerals remain speculative, the occurrence of some nutrient elements has been closely associated with specific components of N_2_-fixing nodules. For example, a low concentration of Mg, S and Ca was found in the inner cortex of soybean nodules formed by *Bradyrhizobium japonicum* strain RCR3442 when compared to strain RCR3407 (Minchin et al. 1994). In another study, P distribution was high in the bacteria-infected region, while K and Cl^−^ were lower in the same component (Mizukoshi et al. 1995). Fernandez-Paschual et al. (1996) also found a low distribution of Cl^−^ in the bacteria-infected zone when compared to the cortex. However, Ca was higher in the outer and inner cortex, but lower in the medulla, of soybean nodules (Mizukoshi et al. 1995). Furthermore, rare elements have been found in tissues of many plants (including legumes), but their functions remain unknown (Tyler 2004; Kastoril et al. 2010).

**Fig. 1:**
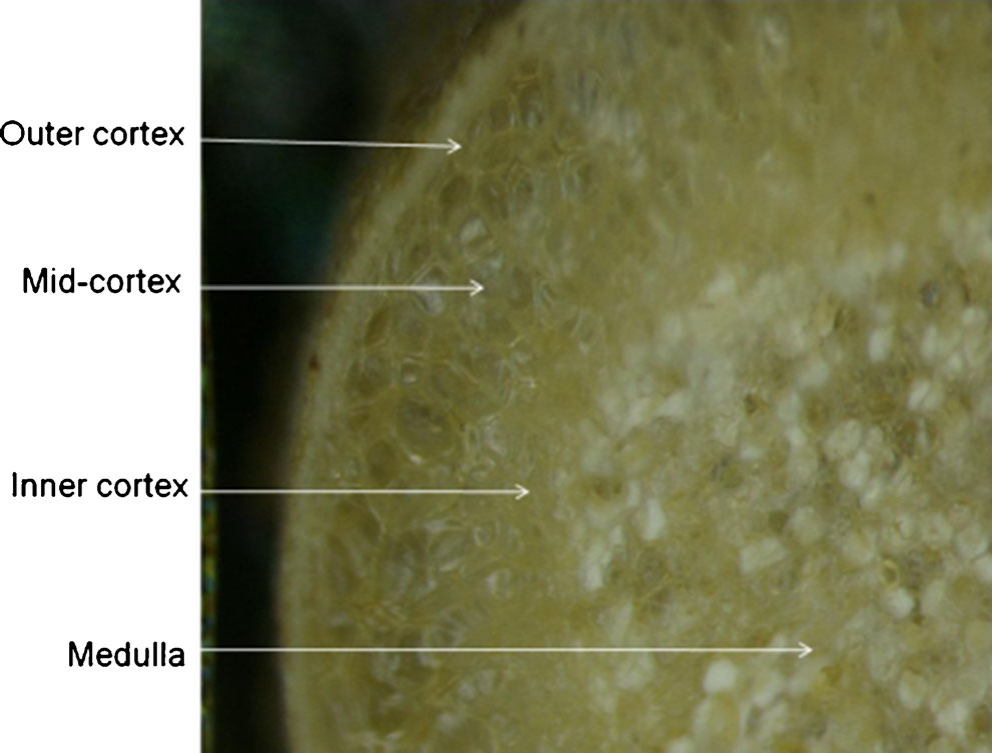
Light micrograph of a medial section of *Psoralea pinnata* (L.) nodule developed naturally in well-drained upland soil. Outer cortex, middle cortex, inner cortex and medulla are shown

*Psoralea pinnata* (L.) is a legume that is adapted to both wetland and upland conditions in the Cape Fynbos of South Africa. It forms effective root nodules in the two differing habitats and derives about 60–88 % of its N nutrition from symbiotic fixation (Kanu and Dakora 2012). *Psoralea* is a member of the tribe Psoraleeae, which is closely related to the tribes Phaseoleae and Desmodieae (Sprent 2009), and exports ureides as the product of N_2_ fixation (Kanu and Dakora 2012). The adaptation of *P. pinnata* to the two contrasting environments (i.e. low pO_2_ in wetland vs. ambient pO_2_ in well-drained upland soils) is intriguing. In this study, particle-induced X-ray emission (PIXE) and backscattering spectroscopy (BS) was used to assess and quantify elemental distribution in different nodule components (i.e. outer cortex, middle cortex, inner cortex and bacteria-infected medulla; see Fig. 1), as well as in the infected and uninfected interstitial cells of the medulla in N_2_-fixing nodules harvested from *P. pinnata* (L.) plants growing under wetland and upland conditions in the Cape Fynbos of South Africa. (see Table 1 for soil properties).

**Table 1:** Elemental composition (soil properties) of *Psoralea pinnata* (L.) rhizosphere soils collected from wetland (Betty’s Bay) and dry-upland (Kleinmond) conditions in the Fynbos of South Africa

Soil property	Wetland (mg/kg)	Upland (mg/kg)
Ca	253.5 ± 53.3a	653.5 ± 199.2a
Mg	147.0 ± 34.5a	49.3 ± 12.4b
K	40.8 ± 1.9a	13.3 ± 1.1b
Na	55.8 ± 10.0a	15.5 ± 2.7b
P	14.5 ± 2.9a	9.3 ± 1.9a
Cu	0.5 ± 0.2a	0.4 ± 0.1a
Zn	1.4 ± 0.5b	8.1 ± 2.4a
Mn	2.3 ± 0.4b	4.69 ± 0.9a
B	0.07 ± 0.01a	0.06 ± 0.02a
Fe	369.3 ± 121.5a	31.73 ± 7.3b
S	14.6 ± 3.5a	3.2 ± 0.7b

Mean (±S.E.) values followed by dissimilar letters in a row are significantly different at *P* ≤ 0.05. The pH (KCl) for wetland and upland soils were 3.45 ± 0.2 and 5.60 ± 0.4, respectively

## Materials and methods

### Plant material

*P. pinnata* (L.) plants were harvested from both wetland and well-drained upland conditions in Kleinmond and inside the Harold Porter Botanical Gardens in Betty’s Bay, Western Cape, South Africa (see Kanu and Dakora 2012). Young *P. pinnata* plants were dug up with their roots and nodules intact from two study sites, and placed in a box containing ice. The plant samples were taken to the laboratory at iThemba Laboratory for Accelerator-Based Sciences (LABS), and mature fully developed nodules removed and thoroughly washed with deionised water. The nodules were blotted dry and photographed before sectioning.

### Soil collection and determination of plant-available minerals

Samples of about 20 g of soil each were collected from the two study sites (four replicates per site) around the roots of *P. pinnata* (L.) plants, air-dried, sieved (2,000 μm aperture), placed in labelled plastic bags prior to analysis. Plant-available minerals in the soil were determined by aspiration on a calibrated simultaneous inductively coupled plasma-mass spectrometer (IRIS/AP HR DUO Thermo Electron Corporation, Franklin, Massachusetts, USA) as described by Makoi et al. (2010).

### Preparation for elemental microanalysis

Fresh nodules were harvested from plants and carefully washed with deionised water. The nodules were hand-sectioned (about 0.6–1 mm thick) with a razor blade under dissecting microscope. Sectioned samples were immediately frozen by immersion in liquid propane cooled by liquid nitrogen using a Leica CFC Cryoworkstation (Leica Microsystem AG, Austria) and freeze-dried for 208 h under vacuum (10^−3^ mbar) in a Leica EM CFD Cryosorption Freeze Dryer (Leica Microsystem AG, Austria) programmed to start at −80 °C and warmed to ambient temperature to prevent water condensation on samples. Such a long cycle was applied in order to minimize shrinkage of specimens. All sectioned nodules were pink in colour due to the presence of leghaemoglobin (an indication of N_2_-fixing effectiveness).

Some of the freeze-dried sectioned nodules were very carefully hand-sectioned under a dissecting microscope to expose the infected and uninfected interstitial cells in the medulla. Each of the processed sections were mounted between two layers of 0.5 % (*w*/*v*) Formvar film coated with a thin carbon layer on the side of the incoming beam to prevent charge build-up during measurements. For easy identification of specimens during irradiation, tissue selection and/or interpretation of micro-PIXE maps, light micrographs of each specimen were captured with a stereomicroscope. The specimens were then stored in desiccators prior to X-ray microanalysis.

### Elemental X-ray microanalysis

Elemental analysis was performed using the nuclear microprobe at the Materials Research Department of iThemba LABS, South Africa. A proton beam of 3.0 MeV energy and 100–400 pA current was focused to 3 × 3 μm^2^ spot and raster-scanned over the section using square or rectangular scan patterns with variable sizes (up to 2.5 mm × 2.5 mm) and variable number of pixels (up to 128 × 128). Particle-induced X-ray emission (PIXE) and proton backscattering spectrometry (BS) were used simultaneously. An external 125-μm Be absorber positioned between the PIXE Si(Li) detector and a specimen was used to shield the detector from backscattered protons and to attenuate X-rays from major light elements. Processing of PIXE data was performed using GeoPIXE II software (Ryan 2000). Quantitative elemental maps were generated using the Dynamic Analysis method. In addition, PIXE and BS spectra were extracted from regions representing nodule components by drawing contours around them. Next, average concentrations from these regions were obtained from PIXE spectra, and BS spectra were used to obtain the specimen thickness and composition of major light elements for matrix corrections. The same procedure was used for single or contiguous groups of infected and uninfected cells within the medulla. Light micrographs of nodule cross-section taken before and after PIXE (especially those of cell shapes/structures and cell arrangement) were used to define the contours of each nodule component. More detailed description of the experimental procedure and experimental setup of the nuclear microprobe can be found elsewhere (Prozesky et al. 1995; Przybylowicz et al. 1999, 2005).

### Histochemical test for the presence of calcium oxalate (CaC_2_O_4_) in nodule cortex: sample preparation and staining

To prepare samples for staining, thin hand-cut sections of freshly harvested nodules from *P. pinnata* were embedded in Technovit 7100 (a hydroxyethyl-methacrylate) according to the manufacturer’s instructions (Kulzer and Co, Wehrheim, Germany) and allowed to cure at room temperature. Semi-thin sections (4–6 μm) were cut from the embedded nodule tissue using a Reichert Ultracut S ultramicrotome system (Reichert-Jung, Austria) fitted with a glass knife, and stained for the detection of calcium oxalate following the procedure of Yasue (1969). Stained sections were examined with a Zeiss Axiocam microscope and photographed.

### Statistical analysis

Element concentrations in nodule components, and in infected and uninfected cells of the medulla, were compared using 1-Way ANOVA, while elemental distribution in components of nodules developed under wetland and upland conditions were compared using 2-Way ANOVA and Duncan test (*P* < 0.05, Statistica v. 8, StatSoft, USA).

## Results

### Elemental distribution in components of N_2_-fixing nodules from *P. pinnata*

A total of 20 elements (Si, P, S, Cl, K, Ca, Ti, Mn, Fe, Ni, Cu, Zn, As, Br, Rb, Sr, Y, Zr, Mo and Ba) were detected in symbiotic nodules from *P. pinnata* plants growing in well-drained upland soils (Table 2). This is the first report on alkali and rare earth elements (Rb, Sr, Y and Zr) being found in N_2_-fixing root nodules of the Leguminosae. With the exception of As and Y, the concentrations of all other elements differed significantly across the nodule components (Table 2). The concentrations of P, K, S, Fe, Si, Mn, Cu and Mo were numerically and/or statistically greater in the bacteria-infected medulla region of upland nodules than cortical components (Table 2).

**Table 2:** Elemental distribution in root nodules of *Psoralea pinnata* (L.) harvested from dry-upland conditions

Element	Elemental concentrations in upland nodules (μg g DW^−1^)			
	Outer cortex	Middle cortex	Inner cortex	Medulla
P	900 ± 90b	800 ± 40b	1,300 ± 170a	1,300 ± 60a
Ca	20,000 ± 900a	2,500 ± 170b	800 ± 40c	1,200 ± 50bc
K	3,100 ± 190b	4,600 ± 50a	4,300 ± 340a	4,900 ± 59a
S	1,400 ± 30c	1,200 ± 60c	2,400 ± 230b	3,200 ± 380a
Cl	1,400 ± 110a	900 ± 20b	800 ± 90b	400 ± 40c
Fe	56 ± 8b	18 ± 1c	41 ± 6b	200 ± 9a
Mo	2.0 ± 0.3b	4 ± 1a	3.0 ± 0.3ab	5 ± 1a
Zn	84 ± 6a	62 ± 2b	58 ± 5b	36 ± 3c
Ni	8 ± 1bc	17 ± 3a	3.0 ± 0.3c	13 ± 2ab
Br	21 ± 2a	5.0 ± 0.4b	4 ± 0.3b	5 ± 1b
Ti	160 ± 20a	13 ± 1b	2.0 ± 0.1b	10 ± 1b
Si	1,500 ± 130a	900 ± 90b	1,300 ± 200ab	1,600 ± 400a
Ba	30 ± 9a	8 ± 1b	10 ± 2b	6 ± 1b
Mn	-	2.0 ± 0.2b	3 ± 1b	26 ± 5a
Cu	14 ± 1a	2.0 ± 0.2b	5.0 ± 0.2b	15 ± 2a
As	4.0 ± 0.4a	4.0 ± 0.4a	4 ± 1a	4 ± 2a
Zr	15 ± 2a	2.0 ± 0.3b	4 ± 0.3b	4 ± 1b
Sr	82 ± 9a	19 ± 1b	12 ± 1b	16 ± 4b
Rb	19 ± 2b	29 ± 4a	26 ± 4a	22 ± 0ba
Y	5 ± 1a	3.0 ± 0.3a	2.0 ± 0.2a	4.0 ± 0.4a

Mean (± S.E.) values followed by dissimilar letters in a row are significantly different at *P* < 0.05

*−* not detected or below detection

The same 20 elements were also detected in wetland nodules (Table 3). The distribution of P, S, Fe, Mo and Si was markedly higher in the bacteria-infected tissue than cortical components, with the levels of P, S, Fe and Mo generally showing an increase from the outer cortex to the medulla region in wetland nodules (Table 3). By contrast, the levels of Cl, K, Ca, Ni, Sr and Zr showed a decrease from the outer cortex through the middle cortex to the medulla region of wetland nodules (Table 3).

**Table 3:** Elemental distribution in root nodules of *Psoralea pinnata* (L.) harvested from wetland conditions

Element	Elemental concentrations in wetland nodules (μg g DW^−1^)			
	Outer cortex	Middle cortex	Inner cortex	Medulla
P	1,700 ± 30c	2,600 ± 110b	2,400 ± 250b	3,400 ± 90a
Ca	14,000 ± 1200a	800 ± 60b	1,300 ± 120ab	1,200 ± 120ab
K	17,000 ± 800a	17,000 ± 1200a	11,000 ± 400b	10,000 ± 100b
S	1,600 ± 190c	1,200 ± 90c	2,700 ± 150b	4,500 ± 170a
Cl	1,500 ± 210a	800 ± 110b	600 ± 60bc	300 ± 50c
Fe	43 ± 4c	40 ± 3c	160 ± 18b	200 ± 20a
Mo	3.0 ± 0.3b	2.0 ± 0.3b	2.00 ± 0.04b	8 ± 1a
Zn	39 ± 3b	71 ± 13a	44 ± 6b	26 ± 2b
Ni	25 ± 2a	4 ± 1b	3.0 ± 0.3b	3 ± 1b
Br	9 ± 1a	3 ± 1b	9 ± 1a	7 ± 1ab
Ti	13 ± 2a	1.0 ± 0.1b	2.8 ± 1.4b	2.0 ± 0.4b
Si	1,300 ± 80b	1,200 ± 100b	1,200 ± 30b	1,600 ± 140a
Ba	41 ± 5a	12 ± 3c	26 ± 1b	6 ± 1c
Mn	39 ± 2a	23 ± 1b	33 ± 3a	37 ± 3a
Cu	5 ± 1a	4.0 ± 0.2a	7 ± 1a	7.0 ± 0.3a
As	4.0 ± 0.1a	2.0 ± 0.1a	2.0 ± 0.1a	2.0 ± 0.1a
Zr	7.0 ± 0.3a	5.0 ± 0.4b	5.0 ± 0.4b	4 ± 1b
Sr	110 ± 13a	37 ± 3b	27 ± 3b	22 ± 2b
Rb	39 ± 1a	29 ± 1b	33 ± 1ab	32 ± 3ab
Y	5.0 ± 0.3a	3.0 ± 0.3b	5.0 ± 0.4a	2.0 ± 0.2b

Mean (± S.E.) values followed by dissimilar letters in a row are significantly different at *P* < 0.05

### Comparison of elemental distribution in wetland vs. upland nodules

Although the concentrations of Si, S, Cl, Ca, Ni, Cu, As, Y, Zr and Ba were unaltered by plant growth under upland or wetland conditions, those of P, K, Mn, Fe, Rb, Sr and Mo increased in wetland nodules, while levels of Ti, Zn and Br decreased (Table 4). The cortical components also showed differences in mineral distribution. The levels of Si, P, S, Mn, Fe and Mo were much greater in the outer cortex of *P. pinnata* nodules, while those of Cl, K, Ca, Ti, Ni, Cu, As, Sr, Y, Zr and Ba showed an increase in the middle cortex (Table 4). After the outer cortex, the nodule medulla was the next component with greater P, S and Fe concentration (Table 4).

**Table 4:** Comparison of elemental concentrations in nodule zones of *Psoralea pinnata* (L.) harvested from upland and wetland conditions

Treatment	Elemental concentrations (μg g DW^−1^)									
A.	Si	P	S	Cl	K	Ca	Ti	Mn	Fe	Ni
Growth condition										
Wetland	1,400 ± 70a	2,600 ± 200a	2,600 ± 300a	790 ± 90a	13,550 ± 630a	4,280 ± 1590a	5 ± 1b	35 ± 2a	110 ± 17a	9 ± 3a
Upland	1,500 ± 70a	1,100 ± 140b	2,200 ± 300a	800 ± 90a	4,226 ± 190b	7,020 ± 2,300a	7 ± 1a	3 ± 1b	84 ± 16b	6 ± 1a
Nodule component										
Outer cortex	1,600 ± 90a	2,700 ± 400a	4,400 ± 110a	400 ± 20c	7,590 ± 1,120b	1,230 ± 27b	3 ± 0b	23 ± 6a	200 ± 7a	2 ± 0c
Middle cortex	1,400 ± 90ab	1,200 ± 150c	1,600 ± 300c	1,400 ± 100a	9,940 ± 2,070a	18,580 ± 2,960a	14 ± 1a	18 ± 6b	39 ± 8c	17 ± 4a
Inner cortex	1,300 ± 100b	1,500 ± 300bc	1,300 ± 100c	810 ± 30b	9,840 ± 1,960a	1,840 ± 410b	5 ± 1b	17 ± 5b	35 ± 6c	7 ± 2b
Medulla	1,300 ± 80b	1,900 ± 300b	2,300 ± 260b	630 ± 50b	8,170 ± 1,270b	950 ± 80b	3 ± 1b	18 ± 5b	113 ± 14b	3 ± 1bc
2-Way ANOVA (F-statistics)										
Growth condition	1.60	123.53***	3.13	0.26	498.87***	4.11	7.00*	431.72***	9.78**	2.09
Nodule component	3.44*	21.73***	48.24***	49.68***	8.04***	40.64***	51.54***	3.93*	90.63***	15.03***
Growth condition × Nodule component	2.73	3.02*	0.34	0.10	12.95***	2.58	2.32	1.84	0.74	10.20***
B.	Cu	Zn	As	Br	Rb	Sr	Y	Zr	Ba	Mo
Habitat										
Wetland	6 ± 0a	37 ± 5b	3 ± 0a	6 ± 1b	36 ± 1a	49 ± 9a	5 ± 1a	5 ± 0a	27 ± 7a	4 ± 1a
Upland	7 ± 1a	54 ± 4a	3 ± 0a	8 ± 2a	21 ± 1b	27 ± 6b	4 ± 1a	5 ± 1a	15 ± 3a	3 ± 0b
Nodule component										
Outer cortex	6 ± 0b	33 ± 3b	3 ± 0ab	6 ± 1b	28 ± 2a	15 ± 2b	3 ± 0b	4 ± 0b	7 ± 1b	7 ± 1a
Middle cortex	9 ± 2a	50 ± 7ab	4 ± 1a	13 ± 2a	28 ± 4a	91 ± 10a	6 ± 1a	10 ± 1a	49 ± 11a	2 ± 1b
Inner cortex	4 ± 1c	54 ± 9a	2 ± 0b	4 ± 1b	31 ± 3a	26 ± 4b	3 ± 0b	3 ± 1b	10 ± 1b	2 ± 0b
Medulla	6 ± 1b	46 ± 6ab	3 ± 1ab	6 ± 1b	29 ± 3a	20 ± 3b	4 ± 1ab	3 ± 1b	19 ± 6b	3 ± 0b
2-Way ANOVA (F-statistics)										
Habitat	3.07	8.01**	0.66	4.18*	66.82***	29.51***	0.65	0.21	4.07	11.57**
Nodule component	26.72***	2.20	2.61	21.46***	0.66	80.72***	3.37*	26.14***	9.93***	37.60***
Habitat x Nodule component	32.67***	1.07	0.86	14.13***	1.55	2.32	1.37	13.24***	0.69	5.86**

Values (mean ± S.E.) with dissimilar letters in the same columns are significant at ***P ≤ 0.001, **P ≤ 0.01 or *P ≤ 0.05

**Fig. 2:**
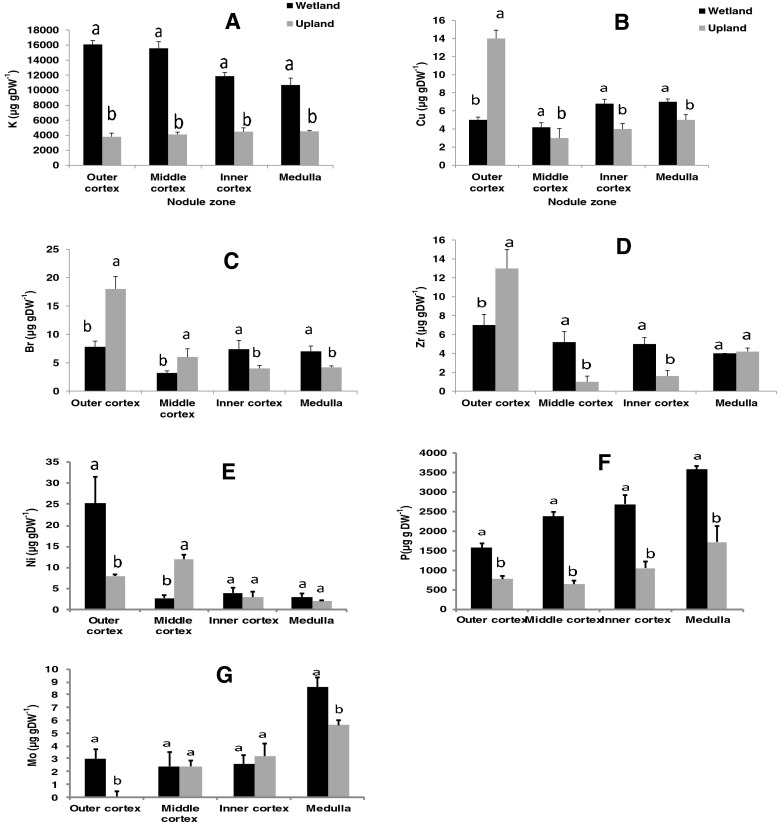
Interactive effects of growth conditions and component of root nodules of *P. pinnata* on concentration of elements (K, Cu, Br,Zr, Ni, P and Mo)

There was a significant habitat x nodule component interaction for P, K, Ni, Cu, Br, Zr and Mo (Table 4). As shown in Fig. 2, the concentrations of K and P were markedly greater in the cortical and medulla region of wetland nodules compared to their upland counterparts. The distribution of Cu and Zn was also greater in the middle and inner cortex of wetland nodules than upland ones, and was the same (Zr) or greater (Cu) in the medulla region of wetland nodules (Fig. 2). The levels of Br, Zr and Cu were markedly higher in the outer cortex of upland than wetland nodules (Fig. 2). There was also a much greater concentration of Mo in the medulla of wetland than upland nodules (Fig. 2).

### Mineral concentrations in infected and uninfected interstitial cells

**Fig. 3:**
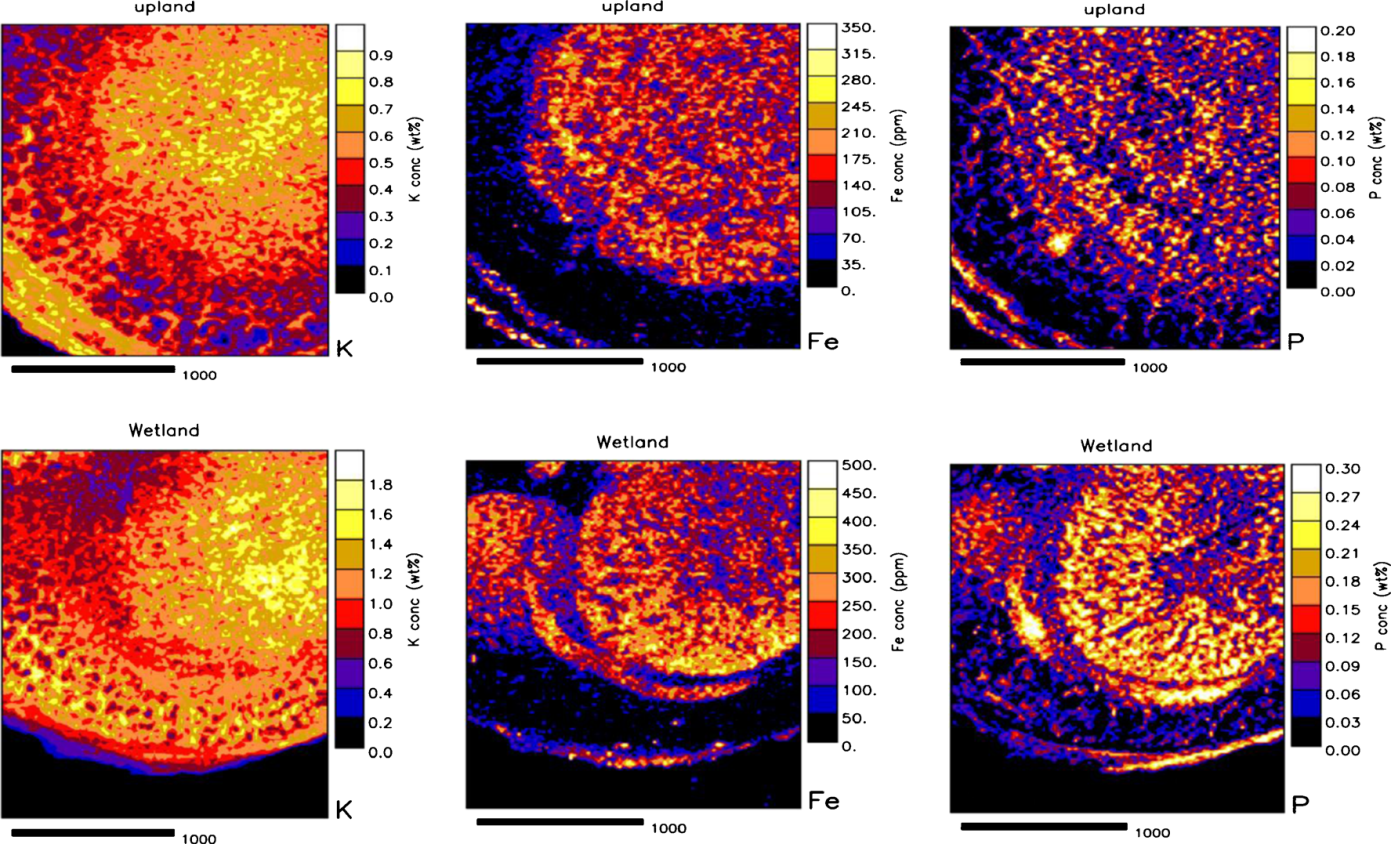
Quantitative elemental maps showing distribution of K, Fe and P in cross-sections of *Psoralea pinnata* (L.) root nodules grown in dry upland (*top*) or wetland (*bottom*) conditions in the Cape Fynbos in South Africa

**Fig. 4:**
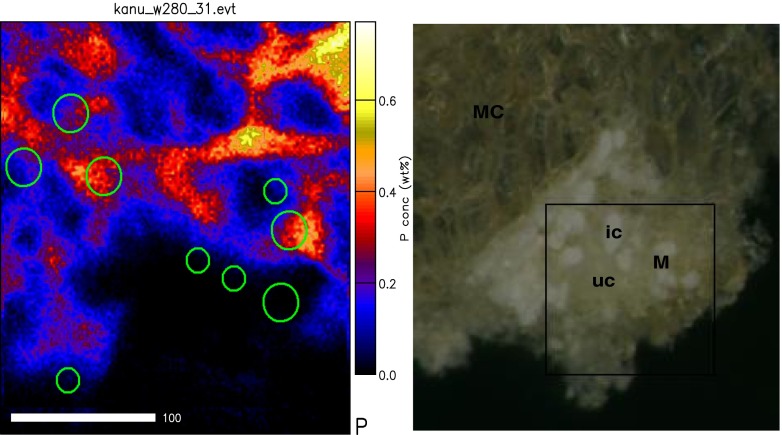
Light micrographs showing marked medulla region (*M*) containing infected cells (*ic*) and uninfected interstitial cells (*uc*) in *Psoralea pinnata* (L.) nodules used for elemental maps (*right*). *Circles* marked on phosphorus map (*left*) represent selected areas from which PIXE and BS spectra were extracted

The distribution of mineral elements in infected and uninfected interstitial cells was assessed in upland and wetland nodules, and As, Rb, Sr, Y, Zr and Mo were found to be below detection limit. A 1-Way ANOVA analysis showed no differences in the levels of K, Ca, Ti, Mn, Ni, Zn and Cu between the two cell types in both upland and wetland nodules (Table 5). There were however significant differences in the levels of Si, P, S, Cl and Fe between infected and uninfected cells of both upland and wetland nodules (Table 5). The distribution of Si, P, S and Fe was much greater in infected cells compared to uninfected interstitial cells of both upland and wetland nodules (Table 5; see also Figs. 3 and 4). In contrast, Cl concentration showed a higher concentration in the uninfected cells of both wetland and upland nodules when compared to infected cells. Although K, Ca, Ti, Mn, Ni, Cu and Zn were also present in both infected and uninfected cells, their concentrations were not significantly different between the two cell types in both upland and wetland nodules.

**Table 5:** Comparison of elemental distribution in infected and uninfected cells in the medulla of root nodules of *Psoralea pinnata* (L.) harvested from both upland and wetland conditions

Elemental concentrations in infected and uninfected cells (μg g DW^−1^)				
Element	Dry-upland		Wetland	
	Infected	Uninfected	Infected	Uninfected
Si	1,700 ± 300a	1,200 ± 200b	1,300 ± 300a	600 ± 100b
P	2,100 ± 300a	1,100 ± 180b	3,200 ± 500a	1,800 ± 200b
S	2,100 ± 300a	1,500 ± 150b	3,100 ± 400a	1,900 ± 200b
Cl	700 ± 200b	1,500 ± 500a	300 ± 20b	600 ± 80a
Fe	200 ± 13a	140 ± 15b	200 ± 18a	120 ± 11b

Values (mean ± S.E.) followed by dissimilar letters in a row are significantly different at *P* < 0.05 for each habitat (i.e. upland or wetland). K, Ca, Ti, Mn, Ni, Zn and Cu were present but were not significantly different. Elements such as As, Rb, Sr, Y, Zr and Mo were below detection limits

A 2-Way ANOVA analysis of minerals in infected and uninfected cells revealed marked differences in the distribution of Si, S and Fe between upland and wetland nodules. While S was lower in cells of upland nodules, by contrast, Si and Fe occurred in greater concentrations in upland nodules (Table 6). At the cellular level, the concentrations of P and Fe were significantly greater in infected cells relative to uninfected cells (Table 6). By contrast, Cl showed a much lower level in infected cells. An analysis of significant interactions revealed no differences in the concentrations of Si, P, Cl and Fe in infected cells of nodules from upland or wetland *Psoralea* plants (Table 6). However, S concentration was higher in the infected cells of nodules from wetland plants. Although the levels of P, S, Cl and Fe were similar in uninfected interstitial cells of upland and wetland nodules, Si concentration in uninfected cells of upland nodules was twice that of uninfected cells in wetland nodules (Table 6).

**Table 6:** Comparison of elemental distribution in infected and uninfected cells in the medulla region of *Psoralea pinnata* (L) root nodules harvested from upland and wetland plants

Treatment	Si	P	S	Cl	Fe
Habitat					
Upland	1,360 ± 170a	2,150 ± 240a	1,700 ± 200b	560 ± 100a	180 ± 20a
Wetland	900 ± 200b	2,480 ± 300a	2,500 ± 260a	420 ± 49a	140 ± 12b
Cell type					
Infected	1,140 ± 200a	2,930 ± 260a	2,210 ± 290a	340 ± 32b	190 ± 19a
Uninfected	1,120 ± 200a	1,710 ± 210b	1,920 ± 180a	630 ± 60a	140 ± 13b
Habitat × cell type					
Infected					
Upland	1,060 ± 130a	2,710 ± 200a	**1,380 ± 200b**	400 ± 52a	210 ± 15a
Wetland	1,230 ± 290a	3,140 ± 500a	**3,030 ± 410a**	290 ± 21a	170 ± 17a
Uninfected					
Upland	**1,660 ± 280a**	1,600 ± 380a	1,930 ± 300a	710 ± 180a	160 ± 20a
Wetland	**5,80 ± 110b**	1,820 ± 200a	1,910 ± 210a	560 ± 77a	120 ± 11a
2-Way ANOVA (F-statistics)					
Habitat	4.37*	0.92	7.74**	1.53	5.34*
Cell type	0.01	12.76**	0.98	7.12*	9.62**
Habitat × cell type	8.03**	0.10	8.31**	0.04	0.07

Values (mean ± S.E.) followed by dissimilar letters in a row are significantly different at *P* < 0.05

### Histochemical detection of calcium oxalate in outer cortex of *P. pinnata* nodules

**Fig. 5:**
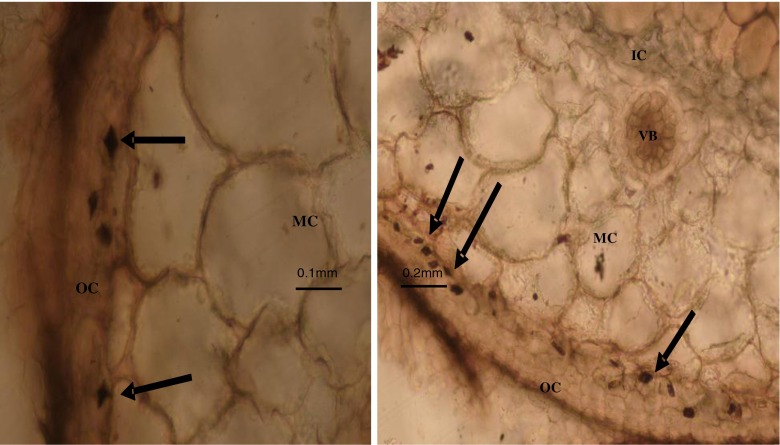
Light micrographs of sections of Technovit-embedded upland *Psoralea pinnata* (L.) root nodules showing the presence of calcium oxalate crystals (*arrow*) in the outer cortex next to the middle cortex cell boundary. Inner cortex (*IC*), middle cortex (*MC*), vascular bundle (*VB*) and outer cortex (*OC*) shown

Microscopic examination of unstained sections of Technovit-embedded nodules revealed the presence of diamond-shaped translucent spaces within the inner portion of the outer cortex (see Fig. 5). These translucent spaces stained black upon treatment with a silver nitrate–dithio–oxamide sequence (i.e. with the Yasue (1969) procedure), indicating a positive reaction for the presence of calcium oxalate crystals in the tissues. X-ray diffraction analysis identified calcium oxalate crystals as whewellite and weddelite in dry powdered samples of *P. pinnata* nodules.

## Discussion

With the legume/rhizobia symbiosis, nutrient elements play a fundamental role in both plant and bacterial metabolism; this includes the synthesis of macromolecules such as leghaemoglobin and bacterial nitrogenase for N_2_ fixation, and chlorophyll for host plant photosynthesis. It is therefore not surprising that the early infection events during nodule formation involve the expression of major symbiosis-related genes (including those for nutrient uptake) in both legume and bacterial partner (Wan et al. 2005; Djordjevic et al. 2003; Rolfe et al. 2003). Some nutrient uptake-related genes activated early during symbiosis include those for siderophore production, phosphate solubilization and ion transporters for phosphate, sulphate, molybdate, iron, zinc, copper and potassium acquisition (Krusell et al. 2005). The expression of these nutrient-uptake genes suggests a metabolic connection between mineral nutrition and symbiotic functioning in nodulated legumes, culminating in the production of ion transporters for supporting N_2_ fixation with essential nutrients.

In this study, micro-PIXE analysis consistently revealed an increase in the distribution of P, S, Fe, Mo and Si in the nodule medulla and infected cells than in the cortex and uninfected interstitial cells of *P. pinnata* nodules (Tables 2, 3 and 4). This is probably not unexpected as many of these elements are components of macromolecules in bacteroids. For example, the formation of nitrogenase requires Fe and Mo for synthesis of the oxygen-sensitive Fe and MoFe proteins of this enzyme (Robson and Postgate 1980; Shaw 1983). Thus, the concentration of Fe and Mo in infected cells, and in the bacteria-infected medulla region of active N_2_-fixing nodules, would be expected to be higher as those elements are required in extra amounts for the synthesis of nitrogenase enzyme. Iron is also needed for the biosynthesis of leghaemoglobin involved in facilitated oxygen diffusion to respiring bacteroids in symbiosomes (Appleby 1984; Appleby 1992; Dordas et al. 2003), and for the synthesis of ferridoxin, an electron carrier in bacteroids.

Bacteroid reactions in symbiosomes also involve various other enzymes that can affect mineral distribution in nodules. For example, ferri-chelate reductase is an enzyme that can contribute to Fe^2+^ concentration in the peribacteroid membrane (Le Vier et al. 1996). Furthermore, the bacteroids in N_2_-fixing nodules also harbour hydrogenases that are either Hup^−^ (if they evolve H_2_ as the end-product of N_2_ fixation) or Hup^+^ (if they oxidize symbiotically-produced H_2_ to yield energy; see Rainbird et al. 1983). Although it is not clear whether *P. pinnata* nodules are Hup^+^ or Hup^−^, both types of hydrogenases are reported to require Fe, S or Ni as building blocks for their subunits (Watt and Ludden 1999). So this, in part, can contribute to the observed increase in S and Fe concentration in infected cells and in the medulla region of *Psoralea* root nodules. Elemental S is also required for cellular construction of the metal S-centres of nitrogenase enzyme (Robson and Postgate 1980; Shaw 1983). So, the higher concentration of S in infected cells and in the bacteria-infected medulla region should be expected as extra amounts of S is needed for nitrogenase synthesis. However, the higher level of S in infected cells of wetland nodules relative to upland nodules (Table 3) can be attributed to the very low concentration of S in upland soil (Table 1).

Silicon is another mineral element that has been found to promote nodule formation and symbiotic functioning in cowpea (Nelwamondo and Dakora 1999). In that study, there was a Si-induced increase in the number of bacteroids and symbiosomes in infected cells, which increased N_2_ fixation (Nelwamondo et al. 2001). So, the higher concentration of Si found in infected cells and in the medulla region of *Psoralea* nodules in this study (Tables 2, 3 and 4) directly confirms its role in symbiotic functioning.

The equally high concentration of P in infected cells and in the medulla of *Psoralea* nodules (Tables 2, 3 and 4) could reflect high rates of oxidative phosphorylation in bacteroids, a process that produces energy in the form of adenosine triphosphate (ATP) for nitrogenase activity. At the cellular level, the products of ATP hydrolysis during N_2_ fixation are adenosine diphosphate (ADP), adenosine monophosphate (AMP) and inorganic P (Pi). These metabolic products (ADP, AMP and Pi) together with unhydrolyzed ATP would be expected to constitute a significant P pool in the cytosol, mitochondria and N_2_-fixing bacteroids of each infected cell (Wei et al. 2004). These can together cause an increase in P accumulation in infected cells and in the bacteria-infected medulla region of symbiotic nodules, as observed in this study (Tables 2, 3 and 4). In fact, the pool size of adenylates (ATP, ADP and AMP) is quite substantial in infected cells, ranging from 45 % in bacteroids to 54 % in both cytosol and mitochondria of infected cells (Wei et al. 2004). Furthermore, these adenylate metabolites have been suggested to act as signals controlling oxygen diffusion in legume root nodules (Wei et al. 2004), operationally aided by the accumulation of K, Ca and P ions in the medulla and inner cortex (or nodule parenchyma) of N_2_-fixing nodules (Minchin et al. 1995). Assuming that is true, the concentration of K, Ca and P would be expected to be high in the medulla and nodule parenchyma, as found in this study (Tables 2, 3 and 4).

Nod factor-induced Cl^−^ efflux during root hair deformation (Felle et al. 1998) is the only known function of Cl^−^ in the legume symbiosis. Yet, in this study, Cl^−^ showed a significantly high concentration in uninfected interstitial cells relative to infected cells (Table 3), and exhibited higher concentration in the nodule outer cortex relative to the medulla, middle or inner cortex (Tables 2, 3 and 4). Elements such as Ti, Sr, Zr and Ba also showed greater accumulation in the nodule outer cortex than the medulla and/or middle/inner cortical regions (Tables 2 and 3). Because this is the first report on the presence of Ti, Rb, Sr, Y and Zr in tissue components of N_2_-fixing nodules, their functions are still unknown. However, Sr^2+^ is reported to replace Ca^2+^ in supporting normal cell growth in symbiotic rhizobia (Humphrey and Vincent 1962).

The presence of Ca^2+^ transporters in symbiosome membrane, the accumulation of Ca^2+^ and nodule-specific calmodulin-like proteins in the symbiosome space during nodule functioning (Krylova et al. 2002; Liu et al. 2006), and the fact that nitrogenase activity decreased with Ca^2+^ depletion in symbiosomes (Krylova et al. 2002) should together suggest greater Ca concentration in the medulla and infected cells of actively-fixing nodules. That was however not the case in this study, greater Ca distribution was found in the cortical region, especially in the outer cortex (Tables 2 and 3).

This increase in cortical Ca was due to the presence of Ca oxalate crystals in the outer cortex (Fig. 5). Other studies (Sutherland and Sprent 1984) have also reported the presence of Ca oxalate in the outer cortex of nodules harvested from *Phaseolus vulgaris*, *Glycine max*, *Vigna mungo*, *Cajanus cajan* and *Vigna radiata* (all ureide-producing legumes), but not in root nodules of *Vicia faba*, *Pisum sativum*, *Lupinus albus* and *Ononis repens* (all amide producers). Because *Psoralea* species export ureides as the product of N_2_ fixation (Kanu and Dakora 2012) and also have Ca oxalate crystals in their nodule cortex (Fig. 5), the presence of Ca oxalate in symbiotic nodules could serve as a taxonomic tool for classifying members of the Phaseoleae.

Whatever the mechanisms of mineral uptake, differences in soil properties appeared to have played a role in the observed distribution of nutrient elements in *Psoralea* nodules. For example, the elements P, K, S and Fe, which occurred in higher concentrations in wetland than upland soils (Table 1), also accumulated in greater levels in tissue components of nodules collected from wetland soils (Tables 2 and 3; Fig. 2). The concentrations of K and P, in particular, were about two- to threefold higher in the outer, middle and inner cortex, as well as in the bacteria-infected medulla region of wetland nodules (see Fig. 2). Thus, the consistently higher distribution of P, K, S and Fe in nodule components under wetland conditions (Tables 2 and 3) could be attributed to the greater availability of these elements in wetland than upland soil (Table 1). Conversely, the high concentration of Ca in upland soil (Table 1) could account for its increased distribution in tissue components of upland nodules (Tables 2 and 3).

Taken together, (a) the greater distribution of mineral elements such as Si, P, S and Fe in infected cells has confirmed their known roles in nodule function, (b) the alkaline earth, alkali and transition elements (Rb, Sr, Y and Zr), never reported before in N_2_-fixing nodules, were for the first time found in root nodules of *P. pinnata* and (c) Cl^−^ (with an unknown function in root nodules) occurred in markedly high concentrations in the uninfected interstitial cells of *P. pinnata* nodules. With the use of genomic tools, it should be possible to determine the role of Rb, Sr, Y, Zr and Cl^−^ in nodule formation and functioning in symbiotic legumes. Hopefully, these findings would become useful only after detailed description of gene expression and metabolic pathways have been done on the effect of the various elements on growth and nodule development. Experiments with qRT-PCR on targeted genes that codify nicotianamine synthase, for example, could unravel why Fe is accumulated and/or preferentially absorbed and transported to infected cells.
